# Neurological Manifestations in Critically Ill Patients With COVID-19: A Retrospective Study

**DOI:** 10.3389/fneur.2020.00806

**Published:** 2020-07-10

**Authors:** Siyuan Fan, Meng Xiao, Fei Han, Peng Xia, Xiaoyin Bai, Huan Chen, Hongmin Zhang, Xin Ding, Hua Zhao, Jing Zhao, Xuefeng Sun, Wei Jiang, Chunyao Wang, Wei Cao, Fan Guo, Ran Tian, Peng Gao, Wei Wu, Jie Ma, Dong Wu, Zhengyin Liu, Xiang Zhou, Jinglan Wang, Tianjia Guan, Yan Qin, Taisheng Li, Yingchun Xu, Dong Zhang, Yu Chen, Jing Xie, Yongzhe Li, Xiaowei Yan, Yicheng Zhu, Bin Peng, Liying Cui, Shuyang Zhang, Hongzhi Guan

**Affiliations:** ^1^Department of Neurology, Peking Union Medical College Hospital, Chinese Academy of Medical Sciences, Beijing, China; ^2^Department of Clinical Laboratory, Peking Union Medical College Hospital, Chinese Academy of Medical Sciences, Beijing, China; ^3^Department of Nephrology, Peking Union Medical College Hospital, Chinese Academy of Medical Sciences, Beijing, China; ^4^Department of Gastroenterology, Peking Union Medical College Hospital, Chinese Academy of Medical Sciences, Beijing, China; ^5^Department of Intensive Care Medicine, Peking Union Medical College Hospital, Chinese Academy of Medical Sciences, Beijing, China; ^6^Department of Respiratory and Critical Care Medicine, Peking Union Medical College Hospital, Chinese Academy of Medical Sciences, Beijing, China; ^7^Department of Medical Intensive Care Medicine, Peking Union Medical College Hospital, Chinese Academy of Medical Sciences, Beijing, China; ^8^Department of Infectious Disease, Peking Union Medical College Hospital, Chinese Academy of Medical Sciences, Beijing, China; ^9^Department of Cardiology, Peking Union Medical College Hospital, Chinese Academy of Medical Sciences, Beijing, China; ^10^School of Public Health, Chinese Academy of Medical Sciences and Peking Union Medical College, Beijing, China

**Keywords:** COVID-19, neurological manifestations, critically ill, stroke, neuromuscular diseases

## Abstract

**Background:** The complications of coronavirus disease 2019 (COVID-19) involved multiple organs or systems, especially in critically ill patients. We aim to investigate the neurological complications in critically ill patients with COVID-19.

**Methods:** This retrospective single-center case series analyzed critically ill patients with COVID-19 at the intensive care unit of Tongji Hospital, Wuhan, China from February 5 to April 2, 2020. Demographic data, clinical and laboratory findings, comorbidities and treatments were collected and analyzed.

**Results:** Among 86 patients with confirmed COVID-19, 54 patients (62.8%) were male, and the mean (SD) age was 66.6 (11.1) years. Overall, 65% patients presented with at least one neurological symptom. Twenty patients (23.3%) had symptoms involving the central nervous system, including delirium, cerebrovascular diseases and hypoxic-ischemic brain injury, while 6 patients (7%) had neuromuscular involvement. Seven of 86 patients exhibited new stroke and 6 (7%) cases were ischemic. A significantly higher prevalence of antiphospholipid antibodies was observed in patients with ischemic stroke than in those without stroke (83.3 vs. 26.9%, *p* < 0.05). Patients with ischemic stroke were more likely to have a higher myoglobulin level, and a lower hemoglobin level.

**Conclusions:** The clinical spectrum of neurological complications in critically ill patients with COVID-19 was broad. Stroke, delirium and neuromuscular diseases are common neurological complications of COVID-19. Physicians should pay close attention to neurological complications in critically ill patients with COVID-19.

## Introduction

The outbreak of coronavirus disease 2019 (COVID-19) began in Wuhan, Hubei Province in December 2019 and has rapidly spread throughout China (1–3). It is caused by a novel coronavirus, severe acute respiratory syndrome coronavirus 2 (SARS-CoV-2) (1), which is similar to the zoonotic SARS-CoV from 2002 and the Middle East respiratory syndrome coronavirus (MERS-CoV) from 2012 (4). In a short time, COVID-19 has spread worldwide. On March 11, 2020, the World Health Organization (WHO) characterized COVID-19 as a pandemic (5, 6).

The clinical spectrum of the SARS-CoV-2 infection, COVID-19, appears to be wide, including asymptomatic infection, mild upper respiratory tract illness, and severe viral pneumonia with respiratory failure and even death (2). Furthermore, various complications beyond the respiratory system, such as acute myocardial injury, acute kidney injury and gastrointestinal complications, have been investigated (2–4, 7–12).

With the increasing number of confirmed cases and accumulating clinical data, neurological complications associated with COVID-19 have been a challenge for clinical management and have generated considerable concern. Recent data from Wuhan, China, reported neurological complications in 36% of 214 COVID-19 patients (13). The neurological manifestations can vary from mild and unspecific symptoms, such as headache and hyposmia, to catastrophic symptoms, including stroke, acute hemorrhagic necrotizing encephalopathy, encephalitis/meningitis and Guillain-Barré syndrome (13–25). However, neurological features of COVID-19 infection in critically ill patients, have not been fully investigated. Herein, we conducted a retrospective study to analyze the neurological manifestations of critically ill patients with COVID-19 in intensive care units (ICU) to explore various pathophysiological mechanisms that could contribute to neurological complications in these patients.

## Materials and Methods

### Participants and Study Design

This is a single-center, retrospective, observational study performed at the Tongji Hospital, Wuhan, China. A designated ICU was established and specialized for critically ill patients with COVID-19 and was managed by the National Medical Team from Peking Union Medical College Hospital, Beijing, China. We retrospectively analyzed patients with COVID-19 who were diagnosed according to the criteria for critically ill patients with confirmed COVID-19 in our ICU from February 5, 2020 to April 2, 2020. All patients included were confirmed cases with positive reverse transcription-polymerase chain reaction (RT-PCR) results for SARS-CoV-2 before admission or positive serological tests for anti-SARS-CoV-2 specific immunoglobulin (Ig) M and G during hospitalization. The diagnosis and classification of disease severity of COVID-19 were made according to Chinese Management Guidance for COVID-19 Diagnosis and Treatment (7th version) (26). Patients who met one of the following conditions were classified as critically ill: (1) Respiratory failure requiring mechanical ventilation (MV). (2) Shock. (3) Patients complicated with other organ failure who required ICU monitoring and treatment.

All individual-level medical information, including demographic characteristics, medical history, clinical, radiological and laboratory findings, treatments and outcome data, were retrieved from the electronic medical records.

This study was approved by the institutional review board of Peking Union Medical College Hospital (No. S-K1151). Written informed consent was waived as this retrospective study was carried out to investigate an emerging infectious disease. The study was performed in accordance with the Declaration of Helsinki.

### Laboratory and Neuroimaging Evaluation

Head CT scans were performed for patients with severe neurological complications after February 28, 2020 using a transport ventilator. Antiphospholipid syndrome (APS) panels, including serum levels of anticardiolipin IgG, IgM and IgA, and anti-β2-glycoprotein 1 (aβ2GP1) IgG, IgM and IgA were determined using a chemiluminescence assay (QUANTA Flash® assays, Inova) according to the manufacturer's instructions. Metagenomic next-generation sequencing of patients CSF samples was performed according to a standard flow, which has been described elsewhere (27, 28).

### Definitions

Lymphocytopenia was defined as a lymphocyte count <1.1 × 10^9^/L. Coagulopathy was defined as a 3-s extension of prothrombin time or a 10-s extension of activated partial thromboplastin time. Delirium was defined according to Diagnostic and Statistical Manual of Mental Disorders, 6th edition. Flaccid paralysis was defined as bilateral paralysis with the loss of muscle tone and absence of tendon reflexes. Stroke was defined as a syndrome of rapidly emerging clinical signs of focal or global disturbance of cerebral function lasted at least 24 h, or detection of cerebral lesions in accordance with vascular origin on neuroimaging examination. Strokes were further verified and classified into ischemic stroke or spontaneous intracerebral hemorrhage based on neuroimaging results. Hypoxic ischemic brain injury is used to describe diffuse brain injury as a result of hypoxia or reduction of oxygen. The outcome is defined as the condition evaluated on April 2, 2020.

### Statistical Analysis

Statistical analyses were conducted using the Statistical Package for the Social Sciences (SPSS) version 17.0 and EXCEL 1810. Data are expressed as medians with the interquartile range (IQR) or means ± standard deviation (SD) according to the distribution. Analysis of variance (ANOVA), Student's *t*-test, or the Mann–Whitney test (non-normal distributions) were used to analyze continuous variables. Pearson χ^2^ test or a Fisher's exact test were used to analyze categorical variables. A two-tailed *p* < 0.05 was considered statistically significant.

## Results

### Demographic and Clinical Characteristics

We finally included 86 critically ill patients with confirmed COVID-19 after excluding 10 patients without available key information, 11 patients with suspected COVID-19, and two patients with a mild or moderate disease course. Of 86 patients, 54 (62.8%) were male, and the mean (SD) age was 66.6 (11.1) years old. The demographic and clinical features of these patients are summarized in Table 1.

**Table 1:** Demographic and clinical findings of critically ill patients with COVID-19.

	**All patients (*n* = 86)**	**Patients without AIS (*n* = 80)**	**Patients with AIS (*n* = 6)**
Age, years, mean ±*SD*	66.6 ± 11.1	66.5 ± 11.5	68.2 ± 2.1
**Sex**			
Male, *n* (%)	54 (62.8)	49 (61.3)	5 (83.3)
**Presenting symptoms**			
Fever, *n* (%)	75 (87.2)	69 (86.3)	6 (100)
Cough, *n* (%)	65 (75.6)	61 (76.3)	4 (66.7)
Myalgia, *n* (%)	15 (17.4)	12 (15.0)	3 (50.0)
Fatigue, *n* (%)^*^	46 (53.5)	40 (50.0)	6 (100)
Headache, *n* (%)	8 (9.3)	7 (8.8)	1 (16.7)
Dizziness, *n* (%)	6 (7.0)	5 (6.3)	1 (16.7)
**PMH**			
Hypertension, *n* (%)	44 (51.1)	41 (51.3)	3 (50.0)
Diabetes, *n* (%)	19 (22.1)	17 (21.3)	2 (33.3)
CAD, *n* (%)	16 (18.6)	14 (17.5)	2 (33.3)
Ischemic stroke, *n* (%)	7 (8.1)	5 (6.3)	2 (33.3)
Intracranial hemorrhage, *n* (%)	4 (4.7)	4 (5.0)	0 (0)
Smoking, *n* (%)	12 (14.0)	11 (13.8)	1 (16.7)
**Complications**			
Arrhythmia, *n* (%)	29 (33.7)	28 (35.0)	1 (16.7)
AF, *n* (%)	16 (18.6)	15 (18.8)	1 (16.7)
Coagulopathy, *n* (%)	49 (57.0)	46 (57.5)	3 (50.0)
AKI, *n* (%)	35 (40.1)	31 (38.8)	4 (66.7)
Liver injury, *n* (%)	34 (39.5)	32 (40.0)	2 (33.3)
Delirium, *n* (%)	11 (12.8)	11 (13.8)	0 (0)
Intracerebral hemorrhage, *n* (%)	1 (1.2)	1 (1.3)	0 (0)
Hypoxic-ischemic brain injury, *n* (%)	2 (2.3)	2 (2.5)	0 (0)
Flaccid paralysis, *n* (%)	5 (6.3)	1 (1.3)	4 (66.7)
Rhabdomyolysis	2 (2.3)	2 (2.5)	0 (0)
**Treatment**			
Antiviral therapy, *n* (%)	67 (77.9)	62 (77.5)	5 (83.3)
**Immunotherapy**, ***n*** **(%)**			
IVIg, *n* (%)	70 (81.4)	65 (81.3)	5 (83.3)
Steroids, *n* (%)	71 (82.6)	67 (83.8)	4 (66.7)
Anticoagulation, *n* (%)^*^	48 (55.8)	42 (52.5)	6 (100)
Aspirin, *n* (%)	10 (11.6)	8 (10.0)	2 (33.3)
Invasive MV, *n* (%)	70 (81.4)	64 (80.0)	6 (100)
ECMO	5 (5.8)	5 (6.3)	0 (0)
CRRT, *n* (%)	16 (18.6)	15 (18.8)	1 (16.7)
**Outcome**			
Death, *n* (%)^*^	55 (64.0)	54 (67.5)	1 (16.7)
Follow-up duration, d, median (IQR)	35.0 (20.6, 43.5)	30.0 (20.0, 39.0)	66.5 (54.8, 69.3)

*COVID-19, corona virus disease 2019; AIS, acute ischemic stroke; SD, standard deviation; PMH, past medical history; CAD, coronary artery disease; AF, atrial fibrillation; AKI, acute kidney injury; IVIg, intravenous immunoglobulin; MV, mechanical ventilation; ECMO, extracorporeal membrane oxygenation; CRRT, continuous renal replacement therapy; IQR, interquartile range*.

* *P < 0.05*.

Most of these patients presented with fever (87.2%) and cough (75.6%). Fifty-six (65.1%) patients presented with at least one type of neurological symptom (headache, dizziness, myalgia, fatigue or hyposmia), including 15 patients with myalgia, 46 patients with fatigue, 8 patients with headache, 6 patients with dizziness, and none patient complaining of hyposmia.

Underlying cardiovascular diseases, including hypertension, diabetes, coronary artery disease, and stroke, as well as smoking were prevalent in critically ill patients with COVID-19, while hypertension was the most common comorbidity and occurred in 44 (51.1%) patients. A total of 12 (14.0%) patients had a past medical history of stroke, including 7 cases of ischemic stroke, 4 cases of intracranial hemorrhage and 1 case of subarachnoid hemorrhage. One patient reported a medical history of myasthenia gravis; and one patient reported a medical history of Alzheimer disease.

The complications of COVID-19 involved multiple organs or systems, including the lymphohematopoietic system, kidney, liver and heart. Coagulopathy was common and occurred in 49 (57.0%) patients. Sixteen (18.6%) patients were complicated with atrial fibrillation during the disease course of COVID-19.

Of the 86 patients, 70 (81.4%) received invasive MV, 5 (5.8%) received extracorporeal membrane oxygenation, and 16 (18.6%) received continuous renal replacement therapy. Most critically ill patients received antiviral therapy (77.9%) and immunotherapy (81.4% received intravenous immunoglobulin and 82.6% received steroids). Forty-eight (55.8%) patients received anticoagulation therapy because of underlying coagulopathy or thromboembolic events. The fatality rate was high; 55 (64.0%) patients died through April 2, 2020 (the median follow-up duration was 35 days).

### Laboratory Findings on ICU Admission

The laboratory findings of the patients are summarized in Table 2. Lymphocytopenia was common and occurred in 77 (89.5%) patients. Lactate dehydrogenase was elevated in 78 (90.7%) patients. Creatine kinase was elevated in 29 (33.7%) patients and myoglobulin elevation was documented in 26 (30.2%) patients. N terminal pro B type natriuretic peptide (NT-proBNP) was elevated in 60 (69.8%) patients, and cardiac troponin I (cTnI) was elevated in 48 (55.8%) patients. D-dimer was elevated in 55 (64.0%) patients. High-sensitive C-reactive protein was elevated in 80 (93.0%) patients. Interleukin−6 was elevated in 66 (76.7%) patients. Twelve of the 32 (37.5%) tested patients were positive upon APS panel testing.

**Table 2:** Laboratory findings on admission in critically ill patients with COVID-19.

	**All patients (*n* = 86)**	**Patients without AIS (*n* = 80)**	**Patients with AIS (*n* = 6)**
WBC count, 10^9^/L, median (IQR)	12.0 (8.7, 17.1)	12.0 (8.9, 17.4)	12.0 (5.2, 17.6)
Lymphocyte count, 10^9^/L, median (IQR)	0.56 (0.36, 0.80)	0.56 (0.38, 0.86)	0.66 (0.25, 0.73)
Platelets, 10^9^/L, median (IQR)	159 (97, 229)	159 (101, 230)	130 (54, 219)
Hemoglobin, g/L, median (IQR) ^*^	122 (99, 134)	123 (104, 136)	95 (90, 107)
ALT, U/L, median (IQR)	27 (18, 43)	27 (18, 43)	22 (11,47)
LDH, U/L, median (IQR)	486 (241, 650)	493 (350, 642)	375 (280, 741)
Creatinine, μmol/L, median (IQR)	75.5 (51.0, 113.5)	72.5 (51.0, 111.2)	96.0 (72.5, 129.0)
Creatine kinase, U/L, median (IQR)	90 (48, 225)	99 (49, 259)	63 (30, 100)
Myoglobulin, ng/mL, median (IQR) ^*^	148.0 (74.1, 365.6)	114.0 (71.2, 365.3)	281.6 (167.0, 443.7)
cTnI, pg/mL, median (IQR)	43.3 (13.8, 270.1)	42.2 (13.1, 300.8)	106.7 (32.6, 235.5)
NT-proBNP, pg/mL, median (IQR)	992 (398, 3,930)	939 (394, 3,771)	3,110 (2,236, 6,895)
LDL-C, mmol/L, mean ±*SD*	1.84 ± 0.78	1.88 ± 0.76	1.34 ± 0.86
Prothrombin time, s, median (IQR)	16.1 (15.1, 17.7)	16.1 (15.1, 18.0)	16.4 (15.0, 17.3)
aPTT, s, median (IQR)	42.8 (37.4, 47.1)	42.1 (37.3, 46.9)	44.4 (40.5, 49.6)
D-dimer, μg/mL, median (IQR)	9.0 (2.8, 21.0)	9.3 (2.8, 21.0)	3.7 (2.6, 12.0)
Procalcitonin, ng/mL, median (IQR)	0.31 (0.14, 0.81)	0.26 (0.12, 0.80)	0.53 (0.28, 1.30)
hsCRP, mg/L, mean ±*SD*	95.8 ± 67.4	94.5 ± 68.5	112.7 ± 53.8
IL-2R, U/mL, median (IQR)	1,090 (638, 1,650)	1,083 (595, 1,445)	1,593 (1,145, 1,921)
IL-6, pg/mL, median (IQR)	60.4 (29.2, 168.2)	59.8 (28.9, 180.3)	69.4 (47.1, 286.5)
IL-8, pg/mL, median (IQR)	28.6 (18.9, 77.3)	29.5 (16.6, 79.1)	28.2 (22.1, 39.5)
IL-10, pg/mL, median (IQR)^*^	10.9 (5.7, 17.1)	11.4 (5.7, 18.9)	6.1 (5.3, 6.3)
TNF-α, pg/mL, median (IQR)	10.3 (6.8, 19.6)	10.1 (6.8, 20.3)	11.8 (7.0, 13.9)
APS panel positivity, n (%)^*^	12 (37.5)	7 (26.9)	5 (83.3)

*COVID-19, corona virus disease 2019; AIS, acute ischemic stroke; WBC, white blood cell; ALT, alanine transaminase, cTnI, High-sensitive cardiac troponin I; NT-proBNP, N-terminal prohormone of brain natriuretic peptide; LDL-C, low-density-lipoprotein cholesterol; aPTT, activated partial thromboplastin time; hsCRP, high sensitivity C-reactive protein; LDH, lactate dehydrogenase; IQR, interquartile range. APS, antiphospholipid syndrome. APS panel included lupus anticoagulant, antibodies against the membrane phospholipid cardiolipin (IgG, IgM, and IgA isotypes) and antibodies against β2GP1 (IgG, IgM, and IgA isotypes)*.

*The normal range for the parameters: LDH, 135–225 U/L; Creatine kinase, ≤ 170 U/L; Myoglobulin, ≤ 154.9 ng/mL; cTnI, ≤ 34.2 pg/mL; NT-proBNP, <486 pg/mL; D-Dimer, <0.5 μg/mL FEU; hsCRP, <1 mg/L; IL-2R, 223–710 U/mL; IL-6, <7.0 pg/mL; IL-8, <62 pg/mL; IL-10, <9.1 pg/mL; TNFα, <8.1 pg/mL*.

* *P < 0.05*.

### Neurological Complications During the Disease Course

Neurological complications involving the central nervous system (CNS) were common, and 20 (23.3%) patients had at least one neurological complication of the CNS (delirium, acute ischemic stroke, intracerebral hemorrhage and hypoxic-ischemic brain injury). Delirium was presented in 11 (12.8%) patients, which was a reason that patients could not tolerate non-invasive MV and were admitted to the ICU for invasive MV. Two patients had hypoxic-ischemic brain injury, and one of these patients received cardiopulmonary resuscitation.

**Figure 1:**
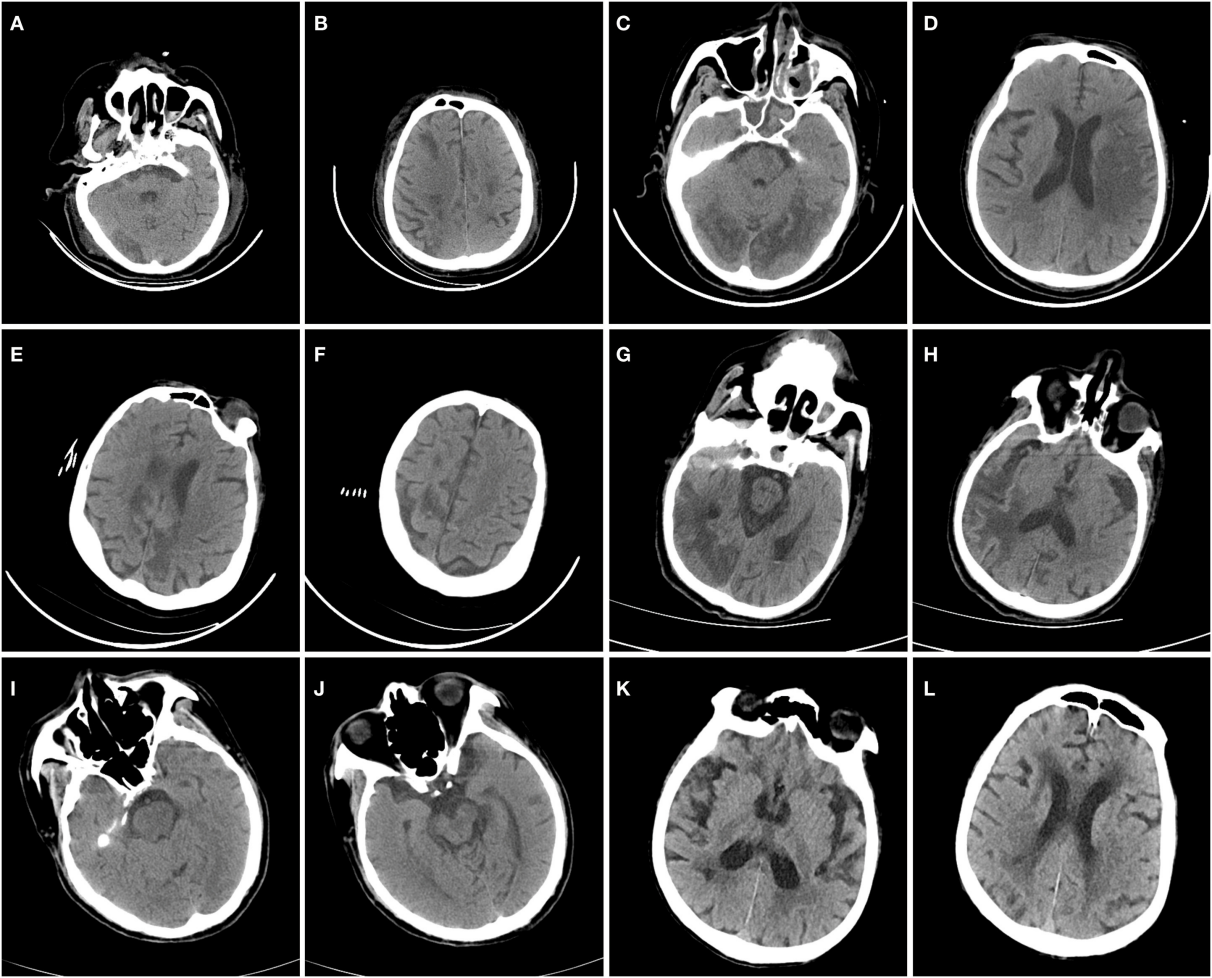
Head CT scans of coronavirus disease 2019 patients with acute ischemic stroke. In Case 1 **(A,B)**, head CT revealed low-density lesions in the right occipital lobe and bilateral frontal and parietal lobes. In Case 2 **(C,D)**, head CT revealed low-density lesions in the bilateral occipital and temporal lobes and the left hemisphere. In Case 3 **(E,F)**, head CT revealed low-density lesions in the bilateral frontal and parietal lobes. In Case 4 **(G,H)**, head CT revealed low-density lesions in the right hemisphere. In Case 5 **(I,J)**, head CT revealed low-density lesions in the left midbrain. In Case 6 **(K,L)**, head CT revealed low-density lesions on the right side of the periventricular area.

Acute ischemic stroke (AIS) occurred in six patients (7.0%, Figure 1, Table 3, Supplementary Figures 1–10), and intracranial hemorrhage occurred in one case. Additionally, two patients presented with acute focal neurologic deficit without neuroimaging evaluation. Of the six patients with AIS, two were deeply sedated, and infarctions were first revealed by head CT in these patients. Five patients were male. Notably, patients with AIS exhibited a significantly higher prevalence of APS panel positivity than those without AIS (83.3 vs. 26.9%, *p* < 0.05). Moreover, patients with AIS were more likely to have a higher myoglobulin level, and a lower hemoglobin level (Table 2). The cTnI and NT-proBNP levels seemed to be higher in patients with AIS, although there was no statistically significant difference between the two groups. All patients with AIS received anticoagulant therapy. Five of six patients with AIS were alive until the end of the follow-up period, and the median survival duration was 66.5 days for these patients.

**Table 3:** Clinical characteristics of COVID-19 patients complicated with stroke^*^.

	**Case 1**	**Case 2**	**Case 3**	**Case 4**	**Case 5**	**Case 6**	**Case 7**
Age range, y	65–70	65–70	65–70	65–70	65–70	65–70	65–70
Sex	Male	Male	Female	Male	Male	Male	Female
PMH	HTN, DM, CAD, stroke^a^	Not notable	HTN, DM, CAD, hyperlipidemia	HTN, stroke^b^, nasopharyngeal carcinoma	MI after COVID-19 onset	COPD	HTN, DM
Diagnosis	Acute ischemic stroke	Acute ischemic stroke	Acute ischemic stroke	Acute ischemic stroke	Acute ischemic stroke	Acute ischemic stroke	Intracerebral hemorrhage
Days after COVID-19 onset when stroke was diagnosed	Day 17	Day 55	Day 32	Day 8	Day 65	Day 19	Day 25
New onset neurological symptoms	Fall, irrelevant answer	Deep sedation, revealed by head CT	Deep sedation, revealed by head CT	LOC	LOC	Slurred speech	Severe headache, LOC
Notable neurological examination	Weakness of four limbs with decreased muscle tone, bilateral Babinski sign (+)	Weakness of four limbs with decreased muscle tone, right Babinski sign (+)	Weakness of four limbs with decreased muscle tone, left Babinski sign (+)	Weakness of four limbs (more severe on the left side), left Babinski sign (+)	Weakness of right limbs, right Babinski sign (+)	Slurred speech	NA (deep sedation after stroke onset)
Head CT	Low density lesions in right occipital lobe and bilateral frontal and parietal lobes	Low density lesions in bilateral occipital and temporal lobes and left hemisphere	Low density lesions in bilateral frontal and parietal lobes	Low density lesions in right hemisphere	Low density lesions in left midbrain	Low density lesions in peri-ventricular area	High density in lateral ventricles and subarachnoid space
Vascular territory	Right PCA, and watershed pattern between right MCA and ACA	Bilateral PCAs, and left MCA	Bilateral ACAs	Right MCA and PCA	Left PCA	Cerebral small vessel disease	-
APS panel	Positive	Positive	Positive	Positive	Positive	Negative	Negative
Outcome	Hosp	Hosp	Hosp	Hosp	Hosp	Died	Hosp

*COVID-19, corona virus disease 2019; CT, computerized tomography; NA, not applicable; LOC, loss of consciousness; PMH, past medical history; HTN, hypertension; DM, diabetes mellitus; CAD, coronary artery disease; MI, myocardial infarction; COPD, chronic obstructive pulmonary disease; APS, antiphospholipid syndrome; PCA, posterior cerebral artery; MCA, middle cerebral artery; ACA, anterior cerebral artery; Hosp, hospitalization*.

* *Some information of case 1, case 3, and case 4 had been reported previously (29)*.

a *Case 1 reported a past history of ischemic stroke and recovered well with modified Rankin score 0 point*.

b *Case 4 reported a past history of ischemic stroke and the previous infarct was located in the cerebellum*.

Neurological complications of the peripheral nervous system and musculature were also observed in critically ill patients with COVID-19. Persistent flaccid paralysis was observed in four patients after withdrawal of sedation. Two patients had rhabdomyolysis.

### Other Notable Neurological Evaluation

Lumbar puncture was performed in two critically ill patients with COVID-19. Protein levels were slightly elevated in one patient with persistent flaccid paralysis, while the opening pressure, white blood cell count and glucose levels were normal. SARS-CoV-2 was not detected in these two patients, either on RT-PCR or on metagenomic next-generation sequencing (Supplementary Table 1).

## Discussion

This retrospective study yields new insight into neurological manifestations in the critically ill patients with COVID-19. Of the 86 critically ill patients with COVID-19 included in this study, 65% presented with at least one neurological symptom. The clinical spectrum of neurological complications in critically ill patients with COVID-19 was broad, including delirium, acute ischemic stroke, intracerebral hemorrhage, hypoxic-ischemic brain injury, flaccid paralysis and rhabdomyolysis. Notably, that cerebrovascular disease was a common comorbidity, and the prevalence of previous stroke in our study was 14%. Moreover, 8% of patients exhibited new stroke during the course of disease, and most strokes were ischemic. Positivity of antiphospholipid antibodies was highly prevalent in patients with ischemic stroke.

CNS symptoms were the main neurological complications in critically ill patients with COVID-19. Only two types of human coronaviruses, namely HCoV-OC43 and E299 were found to be neuroinvasive and can spread from the respiratory tract to the CNS (30). Invasion of CNS by SARS-CoV-2 has been suggested by researchers from the University of Yamanashi, and SARS-CoV-2 RNA can be detected in the CSF of patients with COVID-19 (18). Furthermore, autopsy reports have revealed the presence of virus in neural and capillary endothelial cells in frontal lobe tissue (31), as well as secondary brain damage and neuronal degeneration without evidence of viral encephalitis (32, 33). Recent studies illustrated that COVID-19 has the potential to cause nervous system damage. We performed lumbar puncture in two patients with COVID-19 and neurological manifestations in our ICU; however, neither patient showed signs of significant inflammation in the CSF. Furthermore, RT-PCR assays of the virus and metagenomic next-generation sequencing in the CSF samples were negative. Our findings were consistent with the previous observational report on severe COVID-19 patients, which indicated that RT-PCR assays of the CSF samples were negative for SARS-CoV-2 in all 7 tested patients (23). Whether and how CNS involvement is related to the direct invasion of the virus remains to be addressed in future studies.

Accumulating evidence suggested that neuroimaging features of hospitalized COVID-19 patients were variable, dominated by acute ischemic infarction and intracranial hemorrhages (19–24). Besides, leptomeningeal enhancement, hypoxic-ischemic brain injury, cortical signal abnormalities that may be caused by systemic toxemia were also reported (23, 34, 35). Hypodensities localized in multiple brain areas on CT scans, which were in line with vascular origin were observed in case 1 to case 6 in our series. The lesions of five patients (case 1–5) indicated large artery involvement, while four of them had multiterritory infarcts. Case 6 presented with sudden onset of focal neurological deficit (slurred speech) after admission with a moderate background of cerebral small vessel disease on head CT scan, ischemic stroke of small vessel disease subtype was diagnosed.

Stroke is not uncommon in patients with coronavirus infection. AIS has been reported in patients with SARS and MERS (36–39). To date, 2.3–13.5% of patients with severe COVID-19 have been reported to have comorbid cerebrovascular disease (3, 9). Although stroke has been recognized as a complication of COVID-19 (usually in the severe cases), the exact incidence is not fully investigated (2, 3, 9, 10, 12, 40, 41). Data from Wuhan, China, reported that acute cerebrovascular disease (mainly ischemic stroke) was more common among 88 patients with severe COVID-19 than those with non-severe disease (5.7 vs. 0.8%) (13). In recent case series, ischemic stroke of both large artery- and small vessel- etiology have been reported (19–24). In the present study, stroke was diagnosed in 7 of 86 critically ill patients with COVID-19 and 6 cases were classified as ischemic stroke. This incidence might be higher because neuroimaging examinations were not performed for all patients with acute focal neurologic deficits because of a rapid deterioration of the conditions the result in death. The exact mechanism of ischemic stroke in COVID-19 remains under investigation. Possible explanations include the following.

First, abnormal coagulation results, especially markedly elevated D-dimer and fibrin degradation product, are quite prevalent in critically ill patients with COVID-19, which indicates a common coagulation activation and secondary hyperfibrinolysis condition (42). We also found coagulopathy and antiphospholipid antibodies in critically ill patients with COVID-19 in our cohort (29). Our results indicated that five of the six cases of ischemic stroke had large artery or embolic origin. Similarly, in a previous report of COVID-19 patients with ischemic stroke, all six stroke patients had large-vessel occlusion and three of them had multiterritory infarcts (21). The high incidence of thrombotic complications and the principal subtypes of ischemic strokes verified the existence of a pro-coagulant state in critically ill patients with COVID-19. D-Dimer levels were repeatedly measured in some patients in our study and showed a trend of decreasing, which might be related to anticoagulant therapy. Furthermore, compared with patients without a cerebrovascular event, a significantly higher prevalence of antiphospholipid antibodies was observed in stroke patients. Previous studies have shown an increased risk of developing antiphospholipid antibodies in various viral infections (43). Our results indicate that clinicians should be aware of the increased risk and consider testing for antiphospholipid antibodies in patients with COVID-19 infection and clinical manifestations suggestive of APS. All of the six patients complicated with ischemic stroke received anticoagulant therapy, and five improved or stabilized, which may indicate that critically ill patients with COVID-19 with ischemic stroke may benefit from anticoagulant therapy. Previous studies have also suggested that anticoagulant treatment was necessary and beneficial for severe COVID-19 patients with coagulopathy (44–46).

Second, virus-induced vascular inflammation might be responsible for stroke. In patients with COVID-19, the imbalanced response among T helper cell subtypes could precipitate a cytokine storm syndrome (36). Our results indicated that inflammatory markers were markedly elevated in most critically ill patients with COVID-19. Viral infection and the subsequent immune responses could cause lymphocytic infiltration, necrosis of smooth muscle, endothelial dysfunction and occlusion of large vessel walls. Furthermore, angiotensin-converting enzyme 2 (ACE2), which is a cardio-cerebral vascular protection factor, has been identified as the functional target for SARS-CoV-2 (47). The virus could interact with ACE2 expressed in the endothelium and further attack the vascular system. For patients with underlying cardiovascular disease, SARS-CoV-2 infection can further damage vessel walls through reduction of cerebral blood flow, decreases in oxygen supply and destabilization of arterial plaque. However, we have not been able to demonstrate an association of COVID-19 with vessel wall damage.

Third, there is evidence suggesting that patients with myocardial injury have an increased risk of occurrence of future cerebrovascular events compared with those without myocardial injury (48). Myocardial injury, evidenced by elevated cardiac biomarkers or new electrocardiogram or echocardiographic abnormalities, was recognized among early COVID-19 cases in China (8). In our cohort, more than 50% of critically ill patients had elevated high-sensitivity troponin I and NT-proBNP levels. Myoglobulin level was significantly higher in patients with AIS. Although no statistically significant difference was found because of the small number of stroke patients, higher levels of both cTnI and NT-proBNP levels were observed in patients with COVID-19 with incident ischemic stroke than in those without this event. Furthermore, 18% of the patients were complicated with atrial fibrillation. We speculated that myocardial injury and the concomitant atrial fibrillation may further contribute to the occurrence of ischemic stroke.

Finally, a higher prevalence of anemia was also observed in patients with ischemic stroke in our cohort. Anemia is associated with an increased risk of cerebrovascular events because of decreased tissue oxygen delivery as well as a hyperkinetic state, which disturbs endothelial function and may lead to thrombus formation. Our results indicated that correcting anemia in critically ill patients with COVID-19 might have positive effect on stroke prevention.

In addition to CNS involvement, neuromuscular manifestations, including persistent flaccid paralysis and rhabdomyolysis, were also observed in patients with severe coronavirus infection, which have previously been reported for SARS and MERS (37–39, 49). In a case series study consisting of four patients with SARS who had concomitant neuromuscular problems, the neuromuscular involvement was considered to be critical-illness polyneuropathy or myopathy (50). Significantly elevated inflammatory cytokine levels and immune activation may play a role in neuromuscular injury. We noticed that the prevalence of flaccid paralysis was higher in patient with AIS (66.7%), compared with those without AIS (1.3%). Possible explanations included the longer time at ICU and the more serious clinical conditions for stroke patients. On the other hand, the mortality rate of non-AIS group was high, which limited our ability to withdraw sedatives and determine whether flaccid paralysis exists in these patients. Further electrophysiological and pathological studies are necessary to determine the relationship between COVID-19 and neuromuscular involvement.

Our study has several limitations. First, only 86 patients with confirmed COVID-19 were included in the present analysis, and a large, multi-center study is warranted to verify the neurological manifestations of COVID-19. Second, most of the critically ill patients in our ICU were receiving intensive sedation because of invasive MV, which may have resulted in underestimation of the incidence of neurological complications. Third, some specific information regarding neurological complications, such as brain MRI, imaging evaluations of large intracranial arteries, electrophysiological examinations and CSF profiles were not available. The data were incomplete because of the highly infectious nature of COVID-19, the serious clinical conditions of critically ill patients and the limited conditions for examination in the isolation ward. Thus, we restricted examinations to only those that could have a direct effect on patient management. Fourth, the relatively small number of stroke patients limited the accurate comparisons between patients with AIS and those without. Finally, some patients with neurological complications were still hospitalized at the time of analysis, which may limit the assessment of the ultimate clinical outcome and natural course of the disease, and further long-term observation is needed.

## Conclusions

Stroke, delirium and neuromuscular diseases are major neurological complications of COVID-19. Neurological manifestations might be underestimated in critically ill patients with COVID-19, and physicians should pay close attention to neurological complications. Patients with COVID-19 complicated with ischemic stroke might benefit from anticoagulant therapy.

## Data Availability Statement

The raw data supporting the conclusions of this article will be made available by the authors, without undue reservation.

## Ethics Statement

The studies involving human participants were reviewed and approved by the ethics committee of Peking Union Medical College Hospital (No. S-K1151). Written informed consent for participation was not required for this study in accordance with the national legislation and the institutional requirements.

## Author Contributions

HG and SZ supervised, coordinated and designed the study. SF and FH drafted the manuscript. SF, MX, PX, XB, HC, HZhang, XD, HZhao, JZ, XS, WJ, CW, WC, FG, RT, PG, WW, JM, DZ, and YC collected the clinical data. SF, MX, FH, and JX participated in the interpretation of the data. SF and TG participated in statistical analysis. DW, ZL, XZ, JW, YQ, TL, YX, YL, XY, YZ, BP, and LC supervised and coordinated the study. All authors read and approved the final manuscript.

## Conflict of Interest

The authors declare that the research was conducted in the absence of any commercial or financial relationships that could be construed as a potential conflict of interest.
