# Supplementary material for: Neurological Manifestations in Critically Ill Patients With COVID-19: A Retrospective Study
Source: Front Neurol. 2020 Jul 10;11:806. doi: 10.3389/fneur.2020.00806 (PMC7365850; doi:10.3389/fneur.2020.00806)
Supplement: Supplementary file 1 [file Data_Sheet_1.docx]

Supplementary Material

# Supplementary Figures and Tables

# Supplementary Figures:
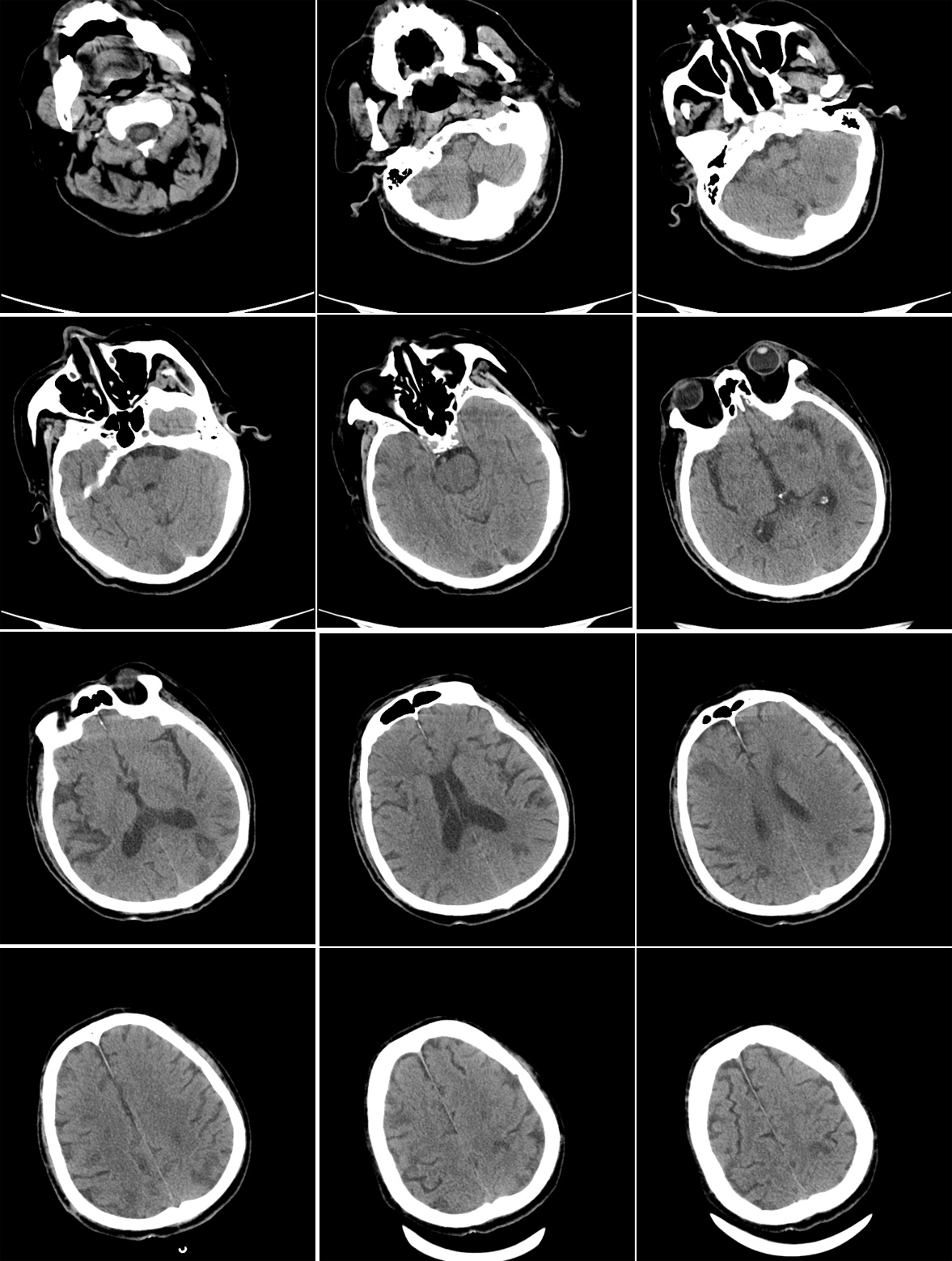


# Supplementary Figure 1: Head CT scan performed 17 days after disease onset in Case 1, which revealed low-density lesions in the bilateral frontal, parietal and occipital lobes.


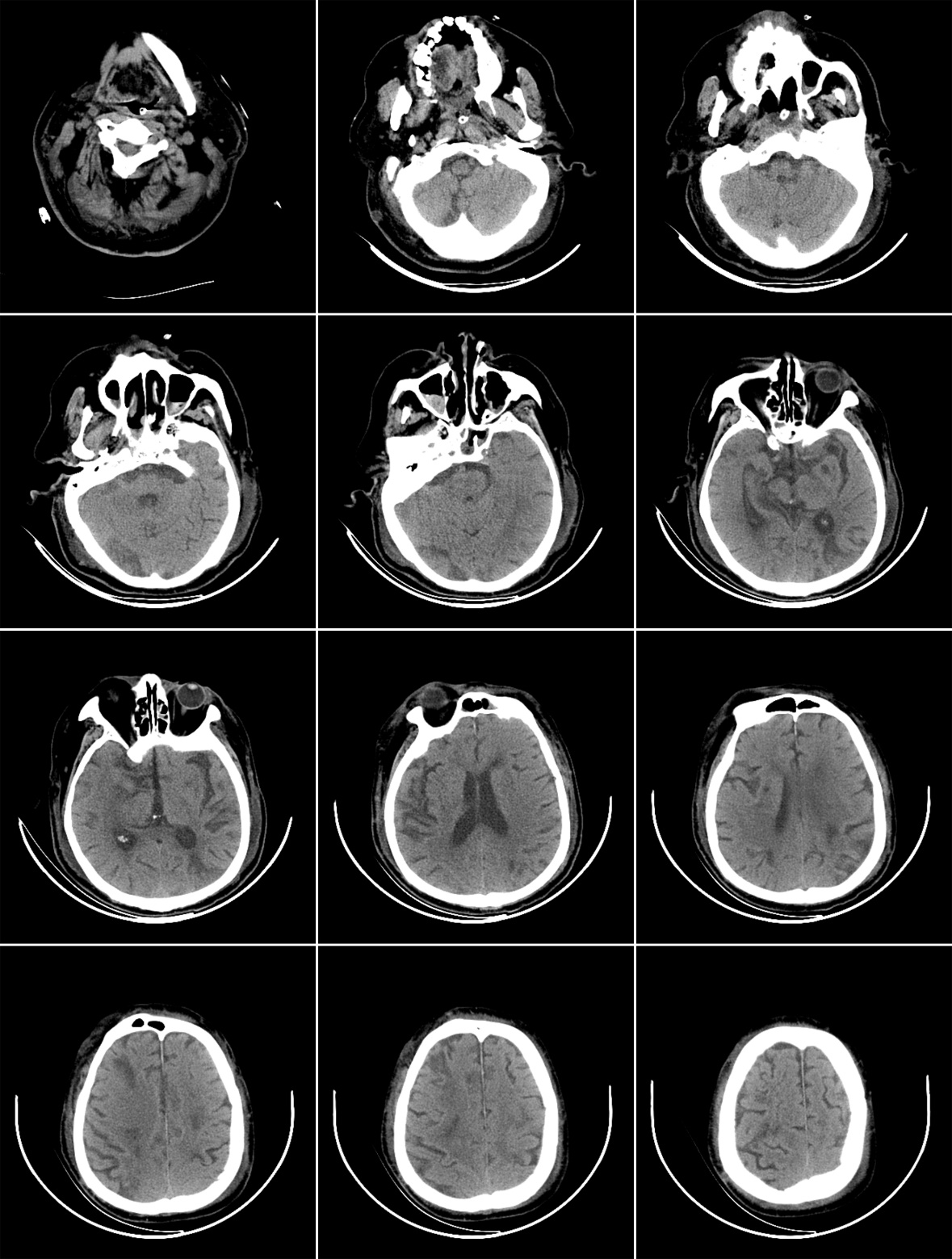


## Supplementary Figure 2: Repeated head CT scan performed 51 days after disease onset in Case 1, which revealed progression of the lesions.


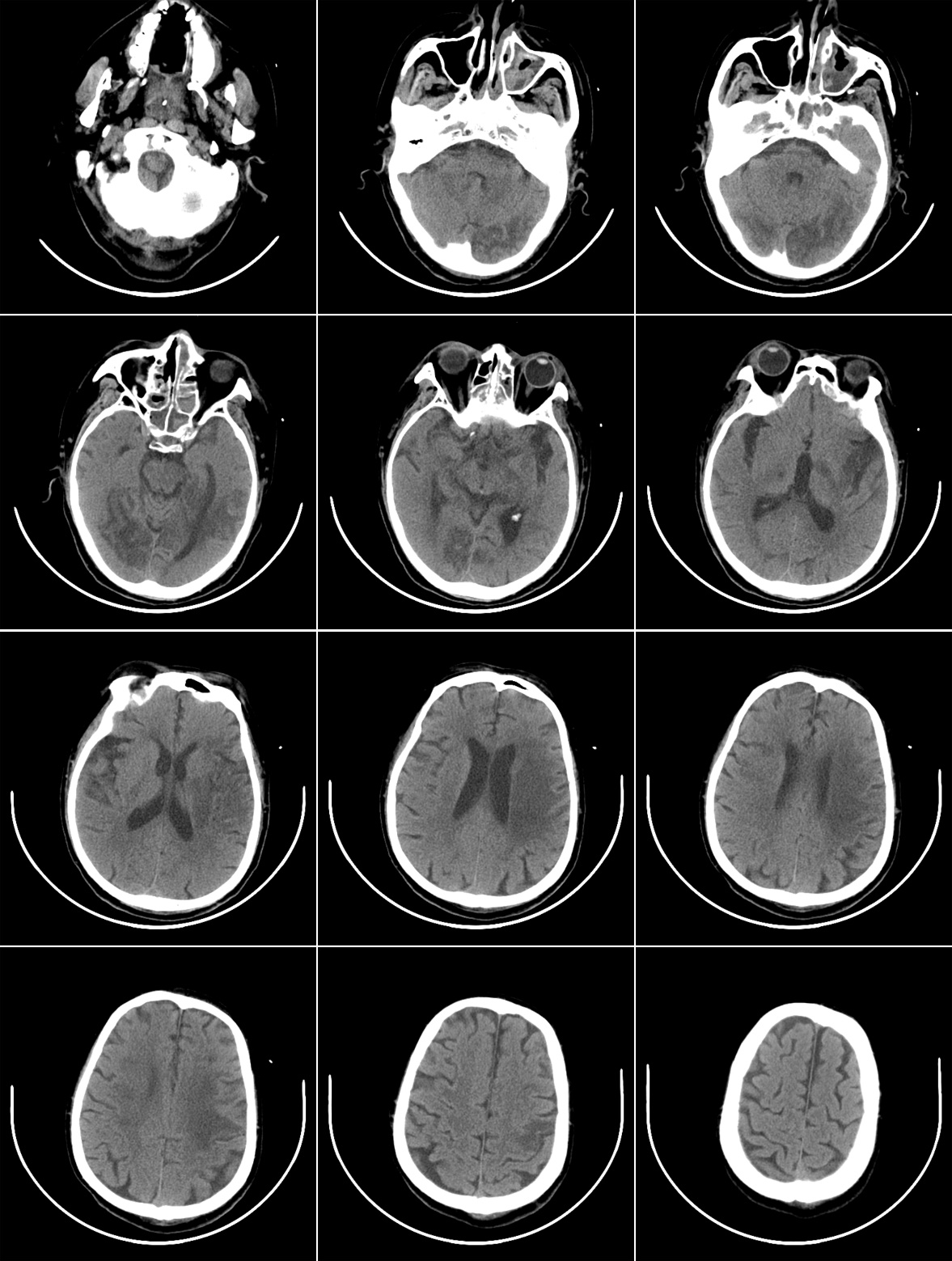


## Supplementary Figure 3: Head CT scan performed 55 days after disease onset in Case 2, which revealed low-density lesions in the bilateral occipital and temporal lobes and the left hemisphere.


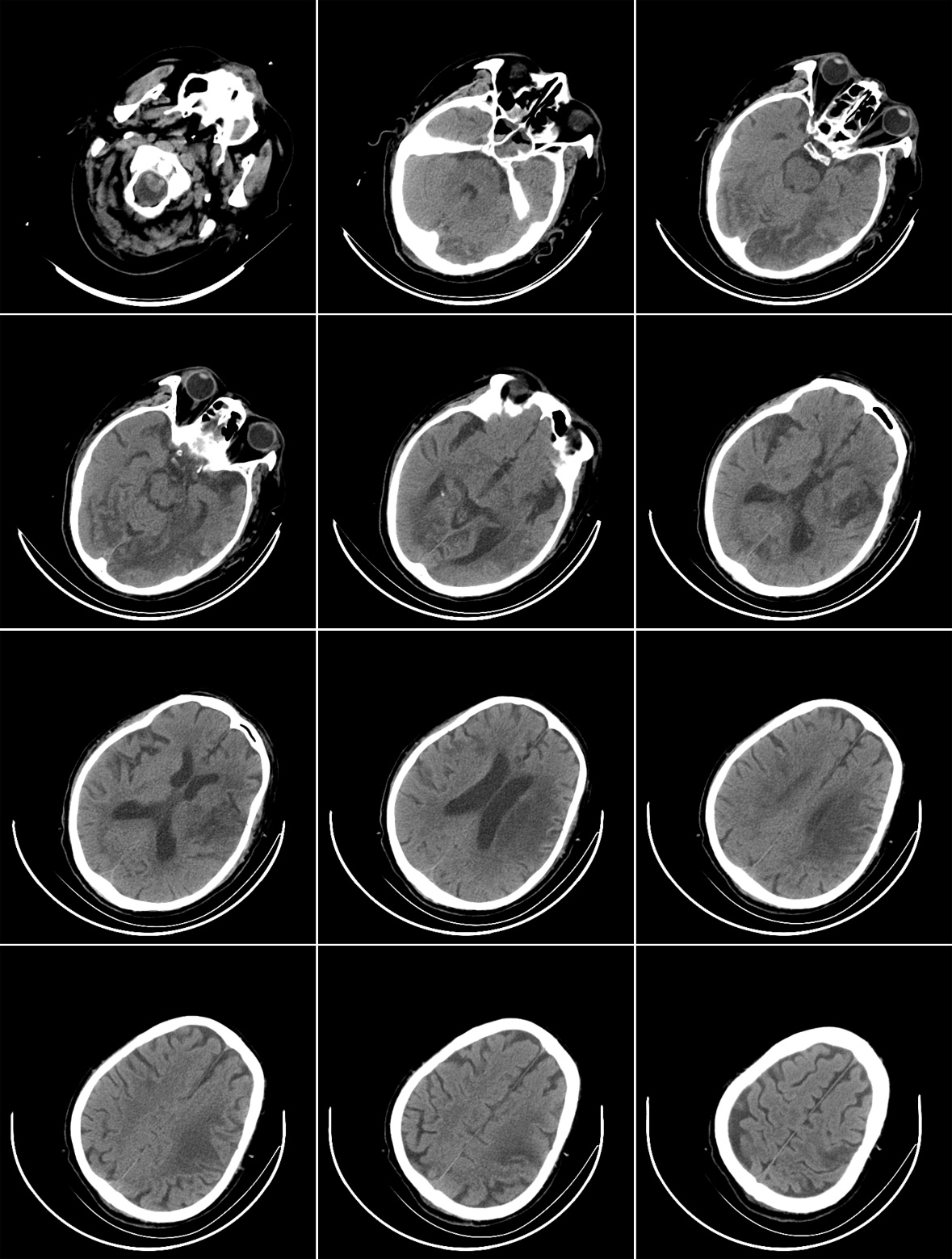


## Supplementary Figure 4: Repeated head CT scan performed 63 days after disease onset in Case 2, which revealed progression of the lesions.


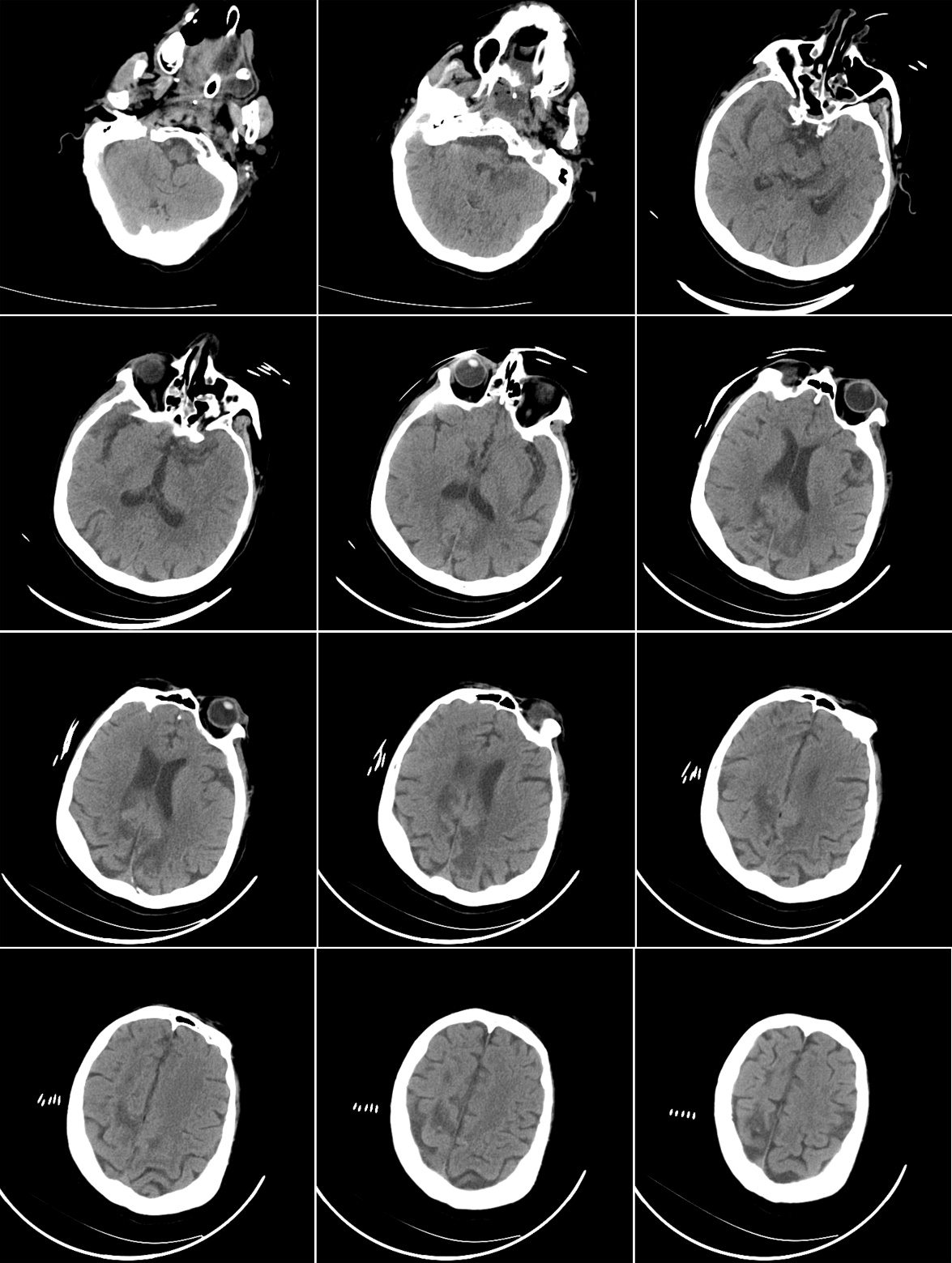


## Supplementary Figure 5: Head CT scan performed 32 days after disease onset in Case 3, which revealed low-density lesions in the bilateral frontal and parietal lobes.


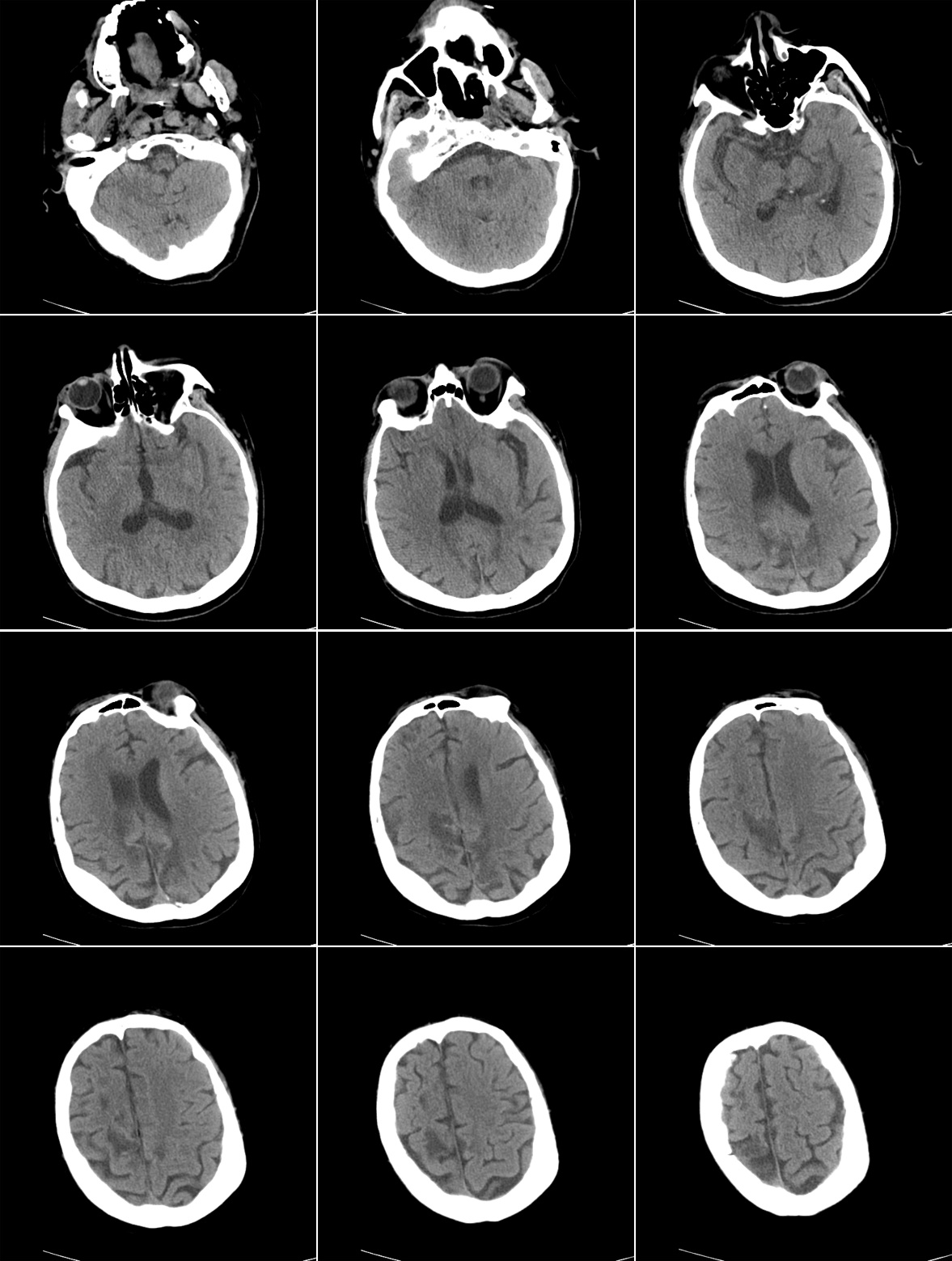


## Supplementary Figure 6: Repeated head CT scan performed 46 days after disease onset in Case 3.


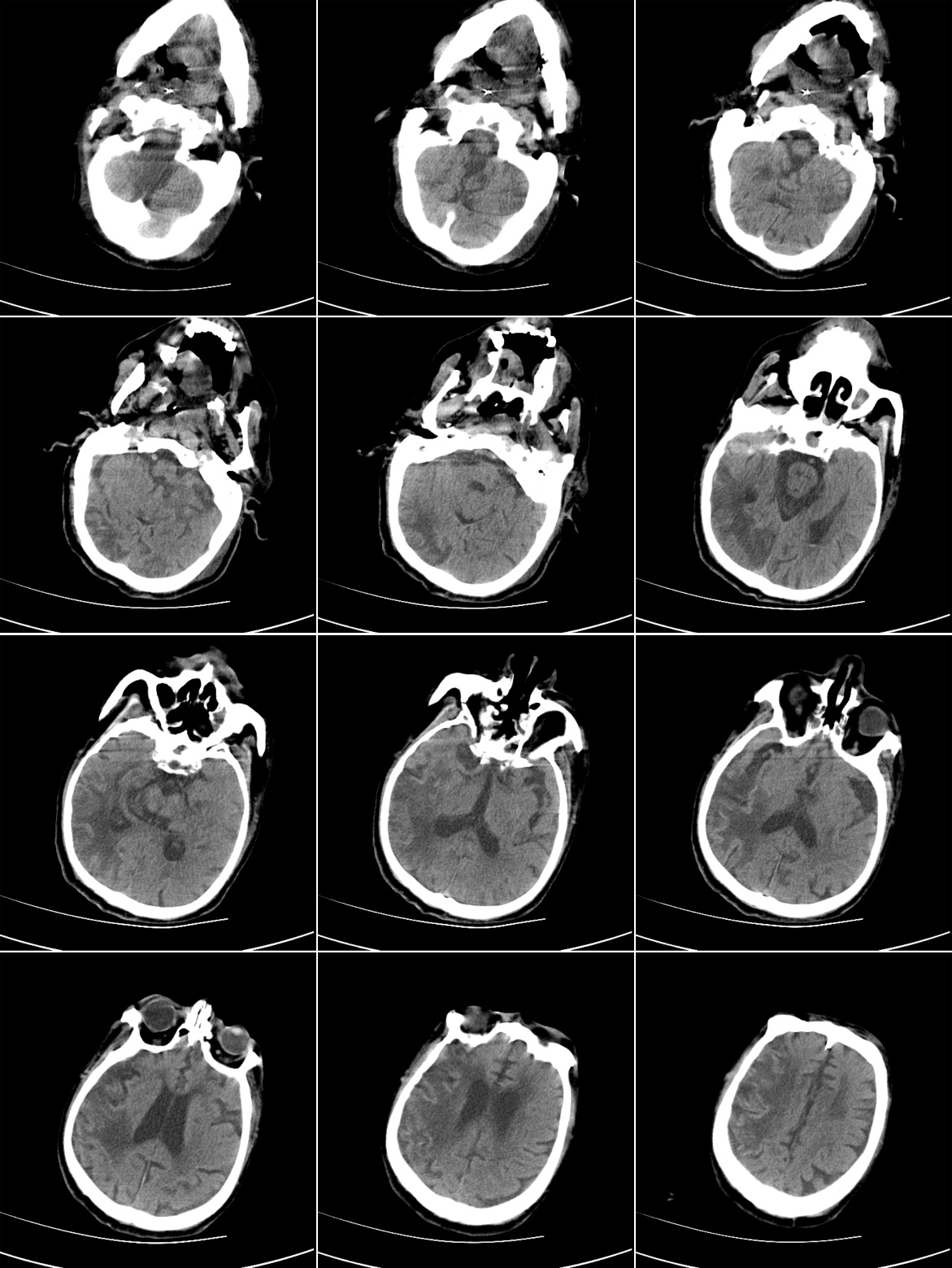


## Supplementary Figure 7: Head CT scan performed 45 days after disease onset in Case 4, which revealed low-density lesions in the right hemisphere.


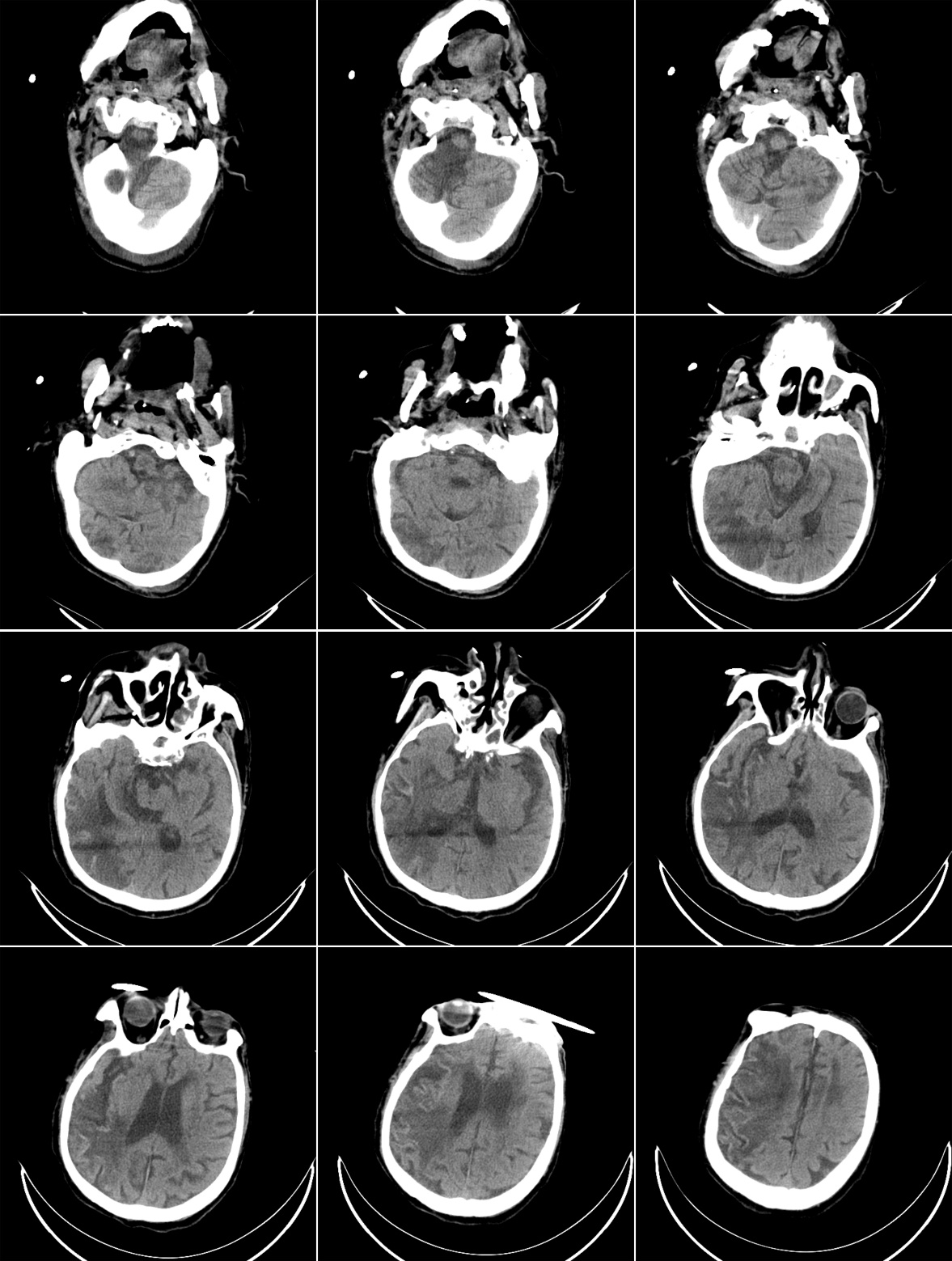


## Supplementary Figure 8: Repeated head CT scan performed 53 days after disease onset in Case 4.

##
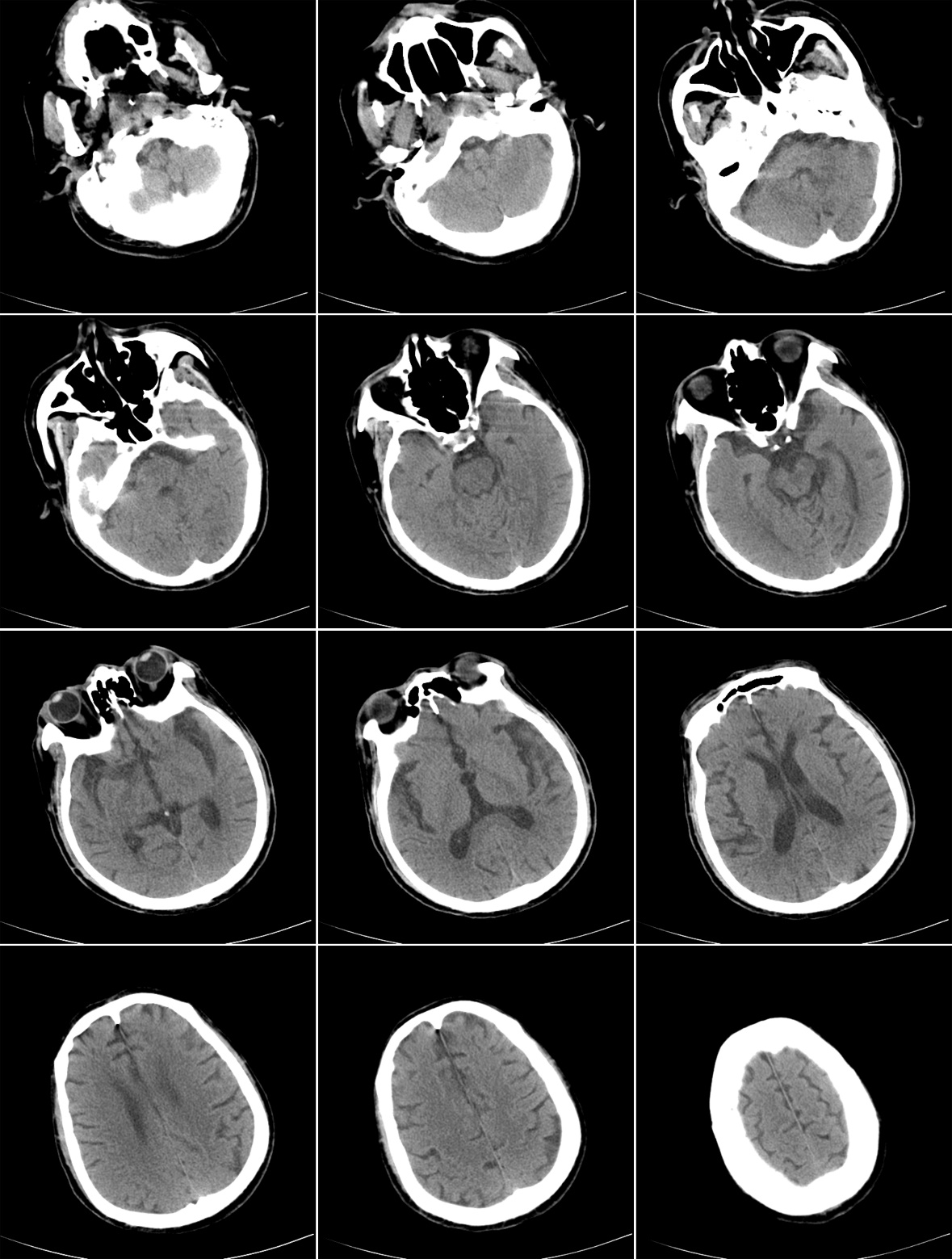


## Supplementary Figure 9: Head CT scan performed 65 days after disease onset in Case 5, which revealed low-density lesions in the left midbrain.


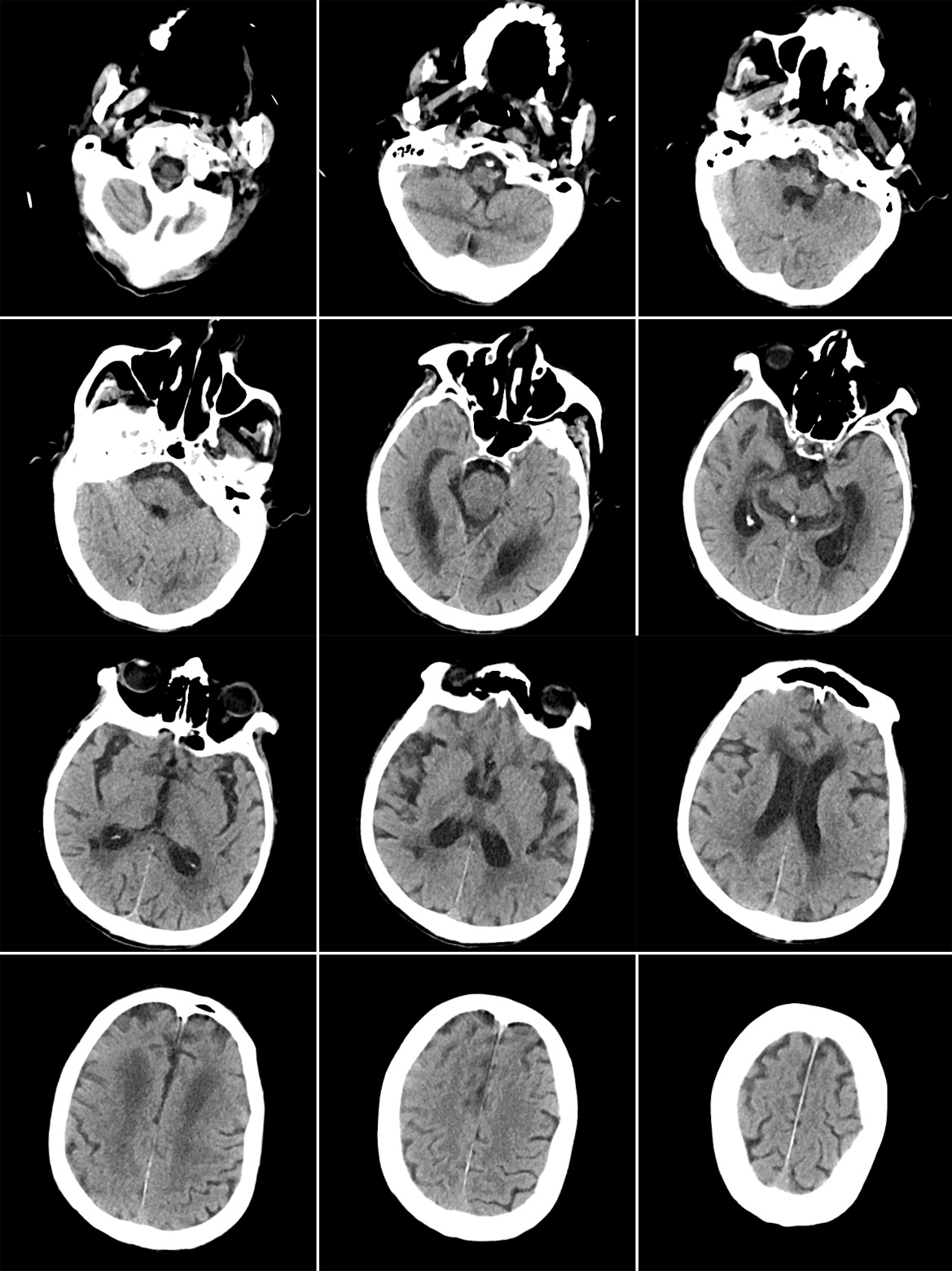


## Supplementary Figure 10: Head CT scan performed 19 days after disease onset in Case 6, which revealed low-density lesions on the right side of the periventricular area with a moderate background of cerebral small vessel disease.

## Supplementary Tables

**Supplementary Table 1.** The CSF results of patients with COVID-19.

|  | Days after COVID-19 onset when LP was perfomed | LP indications | Opening Pressure  (mmH_2_O) | RBC  (10^6^/L) | WBC  (10^6^/L) | Protein  (g/L) | Glucose  (mmol/L) | RT-PCR for SARS-CoV-2 | NGS (DNA and RNA) for virus |
| --- | --- | --- | --- | --- | --- | --- | --- | --- | --- |
| Case 3 | Day 48 | Flaccid paralysis | 60 | 270 | 0 | 0.54 | 4.98 | Negative | Negative |
| Case 10 | Day 28 | Delayed awakening | 144 | 0 | 2 | 0.40 | 6.48 | Negative | Negative |

Abbreviations: COVID-19: corona virus disease 2019; CSF cerebrospinal fluid; LP: lumbar puncture; RBC: red blood cell; WBC: white blood cell; RT-PCR: reverse transcription-polymerase chain reaction; NGS: next generation sequencing; SARS-CoV-2: severe acute respiratory syndrome coronavirus 2.
